# Determination of the Actual Stress–Strain Diagram for Undermatching Welded Joint Using DIC and FEM

**DOI:** 10.3390/ma14164691

**Published:** 2021-08-20

**Authors:** Nenad Zoran Milošević, Aleksandar Stojan Sedmak, Gordana Miodrag Bakić, Vukić Lazić, Miloš Milošević, Goran Mladenović, Aleksandar Maslarević

**Affiliations:** 1 Innovation Center of Faculty of Mechanical Engineering, Belgrade University, 11000 Belgrade, Serbia; mmilosevic@mas.bg.ac.rs (M.M.); amaslarevic@mas.bg.ac.rs (A.M.); 2 Faculty of Mechanical Engineering, Belgrade University, 11000 Belgrade, Serbia; asedmak@mas.bg.ac.rs (A.S.S.); gbakic@mas.bg.ac.rs (G.M.B.); gmladenovic@mas.bg.ac.rs (G.M.); 3 Faculty of Engineering, University of Kragujevac, 34000 Kragujevac, Serbia; vlazic@kg.ac.rs

**Keywords:** actual stress–strain diagram, undermatching weld, martensitic steel, DIC, FEM

## Abstract

This paper presents new methodology for determining the actual stress–strain diagram based on analytical equations, in combination with numerical and experimental data. The first step was to use the 3D digital image correlation (DIC) to estimate true stress–strain diagram by replacing common analytical expression for contraction with measured values. Next step was to estimate the stress concentration by using a new methodology, based on recently introduced analytical expressions and numerical verification by the finite element method (FEM), to obtain actual stress–strain diagrams, as named in this paper. The essence of new methodology is to introduce stress concentration factor into the procedure of actual stress evaluation. New methodology is then applied to determine actual stress–strain diagrams for two undermatched welded joints with different rectangular cross-section and groove shapes, made of martensitic steels X10 CrMoVNb 9-1 and Armox 500T. Results indicated that new methodology is a general one, since it is not dependent on welded joint material and geometry.

## 1. Introduction

The tensile diagram, commonly used in practice, is called engineering stress–strain diagram, with both stress and strain defined with respect to the initial, cross-section *A*_0_ and gauge length *l*_0_. For many engineering problems this approximation is good enough, because stresses and strains are close to their true values, as long as contraction and plastic strains are not significant. Anyhow, in the opposite case, true stress–strain diagram is a better option. In its simplest form, true stress and strains are defined as follows, [1]:

(1) $$\sigma_{t}=\frac{F}{A}=\sigma_{\mathrm{eng}}\left(1+\varepsilon_{\mathrm{eng}}\right)$$

(2) $$\varepsilon_{t}=\frac{\Delta l}{l}=ln\left(1+\varepsilon_{\mathrm{eng}}\right)$$

where *σ*_t_ and *ε*_t_ denote the so-called true stress and strain, respectively, *F* is the acting normal force, *A* current cross-section, which takes into account the contraction, Δ*l* elongation, *l* current referent length, *l* = *l*_0_ + Δ*l*, *l*_0_ initial length, while *σ*_eng_ and *ε*_eng_ denote engineering stress and strain, respectively. It should be noted that terms true stress and strain are used here to emphasize the difference with respect to engineering stress and strain, and should not be understood literally. As a matter of fact, modifications of these equations have been in the focus of many researchers for the past few decades.

To start with, based on the fact that contraction is not the only contribution to true stress, couple of other formulas have been proposed, like the formula for equivalent true stress, as defined by Bridgman, [2]:

(3) $$\sigma_{\mathrm{eq}}=\frac{\sigma_{t}}{C_{B}}$$

where $C_{B}$ is the correction factor:

(4) $$C_{B}=\left[{\left(1+\frac{2R}{a}\right)}^{1/2}ln\left\{1+\frac{a}{R}+{\left(\frac{2a}{R}\right)}^{1/2}{\left(1+\frac{a}{2R}\right)}^{1/2}\right\}-1\right]$$

with a and *R* representing the ligament and the radius of curvature at the site of contraction.

The same approach is used by Ostsemin [3], with a different correction factor *C*_O_:

(5) $$\sigma_{\mathrm{eq}}=\frac{\sigma_{t}}{C_{O}}$$

(6) $$C_{O}=\left(1+\frac{a}{5R}\right)$$

In [3], a procedure is suggested for calculating the correction for neck formation for round and plane specimens made of homogeneous material. Other correction factors were used in [4] for deriving equivalent stress–strain curve with axisymmetric notched tensile specimens, with experimental verification and good agreement with the Bridgman correction at large strains. Another approach is based on equivalent strain, as defined by Scheider [5]:

(7) $$\bar{\varepsilon }=\sqrt{\frac{4}{3}\left(\varepsilon_{x}^{2}+\varepsilon_{x}\varepsilon_{y}+\varepsilon_{y}^{2}\right)}$$

leading to:

(8) $$\sigma =\frac{F}{A_{0}}e^{\left(\varepsilon_{x}\right)}$$

By measuring the mean value of axial strain, formula for the true stress was obtained, [5]:

(9) $$\sigma_{t}=\frac{F}{A_{0}}e^{\left(\bar{\varepsilon_{x}}\right)}$$

One should notice that homogeneous material with rectangular cross-section was analyzed in [4,6], where the tensile properties of FH550 and X80 steels were investigated using rectangular cross-section specimens with different thicknesses, respectively.

Tensile diagrams for welded joints have been determined in [7], using novel methods for determining true stress–strain curves for homogenous materials with rectangular cross-section and weldments with round cross-section. In the first case, the relation between the total area reduction and the thickness reduction was derived, consisting of three parts—geometry function, material function, and basic necking curve. In the latter case the central idea was to force plastic deformation at a notch in the material zone of interest, and to obtain the true stress–strain curve of that material zone from the recorded load versus diameter reduction curve.

The same topic was considered in [8], but for different shape of welded joint, the so-called tailor-welded blank weldment. It was concluded that the predicted strain distributions were in good agreement with the measured ones, thus demonstrating the validity of the proposed experimental method to accurately determine the true stress–strain values of the weldment.

More conventional, notched cross weld tensile testing for determining true stress–strain curves for weldments was considered in [9], whereas a method for determining material’s equivalent stress–strain curve with any axisymmetric notched tensile specimens without Bridgman correction was considered in [10]. Further in [11] the stress–strain relation for the weld metal is determined through experimental investigations of round tensile specimens. The true stress–strain curve was developed by using the modified version of the weighted average method. Yet another overmatched welded joint was considered in [12], where mechanical behavior with planar type laminations in the base metal (BM), heat-affected zone (HAZ), and welding bead (WB) was studied. By using HV data, an equivalent true stress–strain curve in the HAZ was estimated, based on corresponding hardness value obtained from the BM and WB. In [13] a method to determine the mechanical properties for the weldment of two dual phase (DP) steels is discussed. Inverse numerical simulation was used to simulate the indentation tests to determine and verify the parameters of a nonlinear isotropic material model for the weldment. Results are presented for tensile tests on smooth, notched, and notched-welded specimens. It was shown that the yield and tensile strengths of the notched specimens are higher than the strength of the smooth specimens of the base material due to the additional notch stresses. Similar research is presented in [14], where the microstructure, macro and micro-mechanical properties of dissimilar A302/Cr5Mo were investigated by metallographic experiments, tensile and nanoindentation tests. Based on inversion analysis, elastoplastic properties were estimated for parent metal, weld metal, as well as fine and coarse grain heat-affected zones.

In neither case, presented here, material heterogeneity of a welded joint was not taken into account if a weldment cross-section was rectangular at the same time. The only such a case known to these authors is the welded joint with true stress–strain curves obtained in a special iterative procedure for all local zones (base metal—BM, weld metal—WM, heat-affected zone—HAZ), have different properties, as shown in a series of papers, [15,16,17]. Anyhow, the iterative procedure presented in [15,16,17] is not an option here, since it does not lead directly to the result and requires both numerical analysis and experimental testing, not only to verify numerical results, but also to obtain them.

Here, attention is focused to the so-called undermatched welded joint, meaning that the yield stress is lower in a weld metal than in a base metal. One should notice that the plastic strain in undermatched weld metal will appear even with relatively low level of loading, not only due to lower yield stress, but also due to stress concentration, as shown in [18,19]. Once plastic strain becomes significant, cross-section is changed and contraction becomes important, although not the only factor affecting the stress increase. Namely, as it will be shown in this paper, the stress concentration is equally important for this analysis. Therefore, we will use the term actual for the stress–strain diagram exclusively for the case when the stress concentration is taken into account, in addition to contraction.

Toward this aim, one important issue tackled here is the true stress evaluation, which is based on Equation (1), and on contraction values measured by using DIC. As it is shown in this paper, there are significant differences between analytical and measured values of contraction, leading to different true stress–strain curves. For that reason, the term true stress–strain curve is used here for curves obtained by using DIC, whereas the curves obtained by using Equation (1) only are referred to as “true” stress–strain curves. Taking this difference into account, the actual stress–strain curves, as presented here, are based on true stress–strain curves obtained by using DIC, and finally, corrected for the stress concentration.

One should notice that this procedure is a general one, since it will be shown that it does not dependent on welded joint materials and geometry, so it can be applied to overmatched welded joints, as well. Anyhow, since the contraction and plastic strain in that case will be shifted to the base metal, there is almost no practical interest for such an analysis from the point of view of welded joints.

In this work the actual stress–strain diagrams of undermatched welded joints with rectangular cross-section, made of martensitic steel X10 CrMoVNb 9-1 and martensitic armored steel Armox 500T are determined. The goal was to check if different levels of undermatching and different shapes of cross-section, as well as different geometry of welded joint, affect actual stress–strain curve, determined by using formulas proposed in this paper. During the experiment, strains were measured in three dimensions using 3D DIC and software Aramis, to evaluate contraction of rectangular cross-section, i.e., to calculate the current cross section of a specimen, so that true stress–strain diagram can be obtained. Finally, correction for the stress concentration is made, using analytical expressions introduced in [20] and verified by comparison with the results of finite element analysis, but only in the case of one material (Armox 500T) and one geometry (specimen P1-1).

Manuscript structure, after the introduction, comprises materials and methods, results, discussion, and conclusions.

## 2. Materials and Methods

Rectangular test specimens are made of martensitic steel X 10CrMoVNb 9-1 (1.4903–by EN 10216) cut from a pipe, and martensitic steel Armox 500T (SSAB, OxelÖsund, Sweden), cut from a plate. In both cases, a combination of TIG and MMA welding process was used for pipe and plate welding. In both cases S Ni 6082 (EN ISO 18274) was used as filler material for the root and hot pass, and filler material E 19.12.3 Nb R 26 (ISO 3581) was used for filling passes. Chemical compositions of base and filler metals are shown in Table 1 and Table 2, respectively.

From Table 1 it can be concluded that the base metals used, although both of martensitic microstructure, have significantly different chemical compositions. This is the case because the martensitic microstructure is not obtained in the same way. For 1.4903 steel, martensite was achieved by alloying and consequent heat treatment, whereas for Armox 500T increased carbon content was used, as well as the heat treatment. Materials will not behave in the same way under loading, and this can be concluded by comparing the mechanical properties presented in Table 3 for the base metals and in Table 4 for the filler metals. Materials with different mechanical properties are used to find out if undermatching level affects the proposed formula for stress evaluation. Namely, as one can see from Table 3 and Table 4, the undermatching coefficient, defined the ratio between weld metal and base metal yield stress (R_p0,2_), is significantly different, circa 0.9 for steel 1.4903 (400/450) and circa 0.32 (400/1250) for Armox 500T.

**Figure 1:**
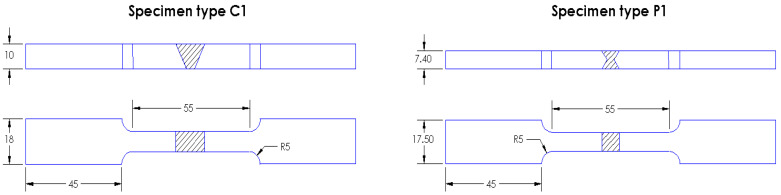
Dimensions [mm] of the specimens for steel 1.4903 (C1) and for Armox 500T (P1).

Test specimens were made with “V” joint for 1.4093 steel and with “X” joint for Armox 500T, as shown in Figure 1. Dimension ratios for C1 specimens (steel 1.4903) are 8/10 = 0.8, and for P1 specimens (Armox 500T) are 7.4/7.5 = 0.99, which is practically square. Different shapes of the specimen cross-sections and grooves are also used to find out eventual effects of welded joint geometry on the proposed formulas for stress evaluation.

**Figure 2:**
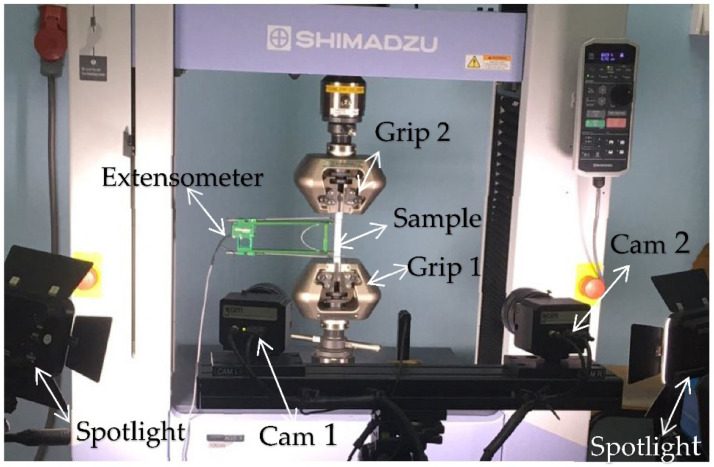
Setting up an experiment with the position of the cameras and with the extensometer set.

Digital image correlation (DIC) is a powerful non-contact technique for measuring surface displacement/strain fields, [21]. Simple geometric shapes can be treated by 2D analysis, while more advanced, 3D analysis, should be used for more complex geometric shapes, including welded joints, as applied and presented in [22,23,24]. The force during the experiment was controlled by strain, with the rate 2 mm/min. Setup of the experiment with the position of cameras is shown in Figure 2. Using DIC method with two cameras (3D deformation measurement) and the Aramis software (Version 2M, GOM GmbH, Braunschweig, Germany) the current cross-section area can be determined. Accuracy of this method for strain measurement is very high, in order of micrometers, so it is a suitable method for the experiment performed here.

Finite element method (FEM) is nowadays a widely accepted numerical tool to get stress and strain distribution for many engineering problems, including elastic-plastic analysis of welded joints, even in the presence of cracks, and for other complex problems, [25,26].

**Figure 3:**
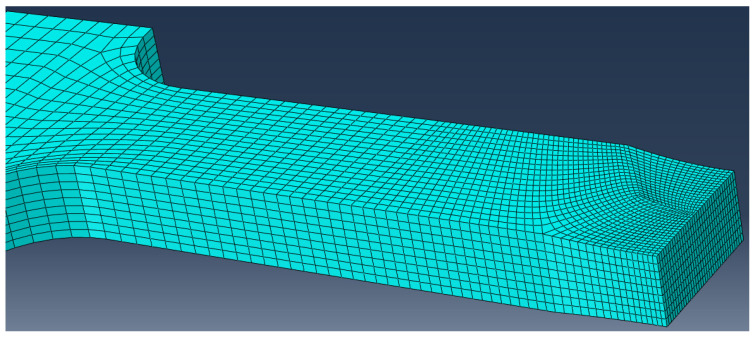
FE model of specimen P1-1 with deformed weld metal.

Here, 3D FEM is used to evaluate stress concentration. Mesh was made with 3D linear elements, C3D8, with 8 nodes, with decreasing size in the weld metal down to 0.4 × 0.2 mm, as shown in Figure 3, where one example of meshes deformed in weld metal is given. One quarter of specimen was modeled due to two planes of symmetry and appropriate boundary conditions applied (one rotation and two translations fixed). Load is defined as the negative pressure, according to the force applied and remote cross-section. More detailed description is given in [20].

## 3. Results

**Figure 4:**
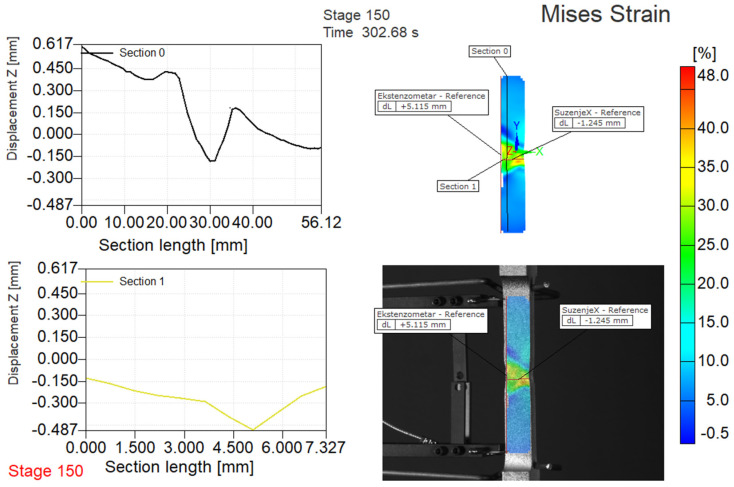
Analysis of changes in the characteristic dimensions of the specimen C1-1.

Typical result for strain measurement by DIC is shown in Figure 4, as obtained by the post-processing, using software Aramis.

**Figure 5:**
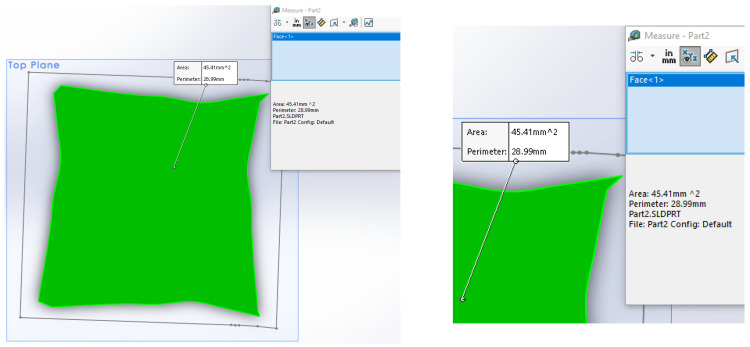
Initial (marked by line) and final (green) cross-section area of specimen P1-1.

The current cross-section area of the specimen was calculated using data obtained by Aramis, as shown in Figure 5 for specimen P1-1. One of the sides was actually measured, the opposite one taken as the mirror image, and two remaining are obtained by rotating the measured one for 90° and −90°.

**Figure 6:**
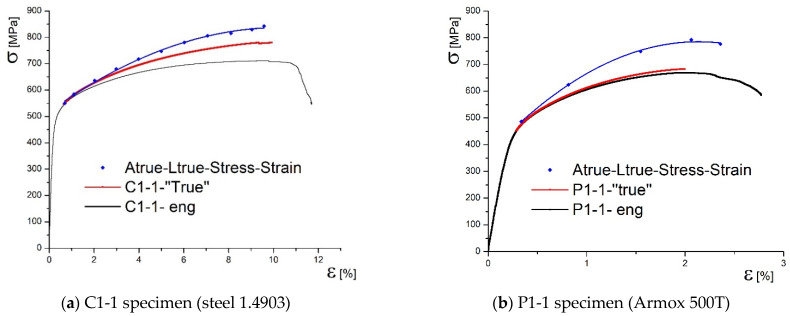
Comparison of true and engineering diagrams calculated by DIC method.

Figure 6 shows three stress–strain diagrams for both specimens, types C1 and P1, including engineering diagram, obtained by standard tensile test, marked in black. Remaining two diagrams represent true stress–strain curves, one determined according to Equations (1) and (2), marked in red, and the other one determined using measured cross-section areas of the specimen by DIC, marked in blue. One can see that the true stress is increased, if contraction measured by DIC (Figure 5) is taken as relevant. This is why red curves in Figure 6 are marked as “true” and blue ones as true.

**Figure 7:**
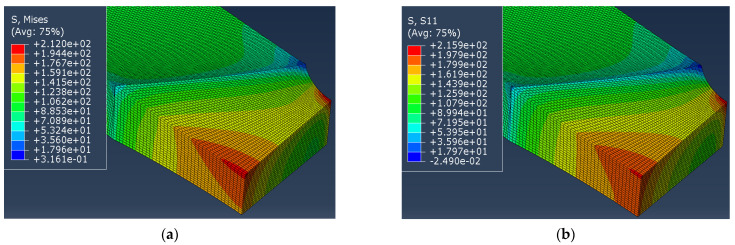
Stress distribution in specimen C1-1 with deformed weld metal: (**a**) equivalent stresses, (**b**) normal stresses, given in MPa.

Results of FEM calculation are shown in Figure 7 for specimen C1-1 as an example of the procedure applied. Results for C1-1 specimen, with deformed weld metal according to strains and contraction obtained by DIC, show equivalent stress distribution, Figure 7a, and normal stresses distribution, Figure 7b, for the applied load 4 KN, producing remote tensile stress 100 MPa in the narrow part of the specimen, away from the welded joint area.

From Figure 7 it can be concluded that the difference between maximum Misses equivalent stress and maximum normal stress is just 3.91 MPa (215.9–212 MPa) or 1.84%. This leads to the conclusion that the equivalent stress is not the dominant parameter for stress increase, but it is rather the stress concentration due to contraction. To calculate the actual stress with the stress concentration taken into account, the authors propose the following equations:

(10) $$\sigma_{\max}^{\mathrm{actual}}=\sigma_{T}C_{\mathrm{NM}}$$

where *C*_NM_ is the stress concentration factor and $\sigma_{T}$ is calculated as:

(11) $$\sigma_{T}=\frac{F}{A_{\mathrm{current}}}$$

**Figure 8:**
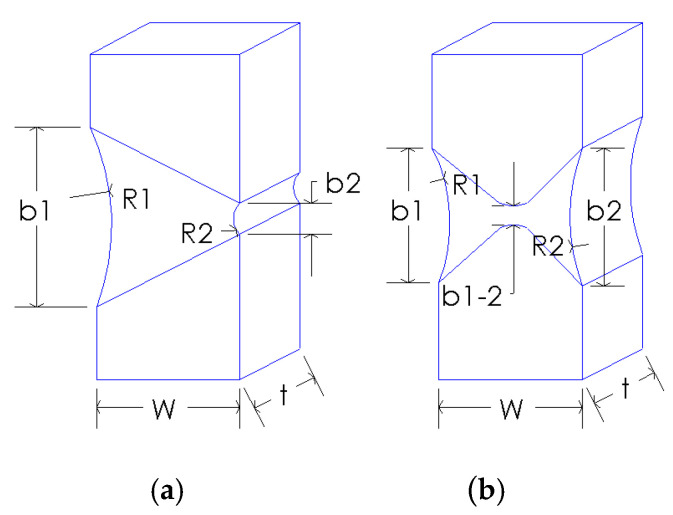
Characteristic dimensions of weld metals: (**a**) V shape, (**b**) X shape.

Stress concentration factor *C*_NM_ can be separated into two factors, as follows:

(12) $$C_{\mathrm{NM}}=C_{\mathrm{ZS}}+C_{\mathrm{EP}}{}_{\;}$$

where *C*zs takes into account the welded joint geometry and *C*_EP_ stands for reduction of thickness. According to [22], *C*zs can be expressed for point 1, as follows:

(13) $$C_{{\mathrm{ZS}}_{1}}=1+\frac{b_{1}}{2\left(R_{1}+b_{1}\right)}$$

where *b*_1_ and *R*_1_ are defined in Figure 8 for two characteristic points in a weld metal, together with their counterparts, *b*_2_ and *R*_2_, used for calculating *C*zs for point 2.

Likewise, *C*_EP_ can be defined as, [22]:

(14) $$C_{\mathrm{EP}}=\frac{\Delta t}{2W_{0}}=\frac{\;\Delta t/t_{0}}{2W_{0}/t_{0}}$$

where *t*_0_ and *W*_0_ are initial values of thickness *t* and width *W*, Figure 8. Therefore, the final expression for the stress concentration factor is:

(15) $$C_{\mathrm{NM}}=\left(1+\frac{b}{2\left(R+b\right)}+\frac{\Delta t}{2W_{0}}\right)$$

The current cross-sectional area of the specimen (*A*_current_) was calculated using the data obtained by the DIC.

In the further analysis, numerical verification of coefficients for specimens C1-1 and P1-1 is shown for strains immediately before the fracture:

- Specimen C1-1

C1-1$t_{0}=8$ [mm]$W_{0}=10$ [mm]F=48342.18 [N]$A_{\mathrm{current}}=63.23293$ [mm^2^]$\sigma_{T}=764.50957$ [MPa]Δt=1.0487 [mm]Point 1$b_{1}=18.866$ [mm]$R_{1}=58.0536$ [mm]$C_{{\mathrm{NM}}_{1}}=1.17507091$$\sigma_{\max_{1}}^{\mathrm{actual}}=\sigma_{T}C_{{\mathrm{NM}}_{1}}=898.353\;\;\left[\mathrm{MPa}\right]$Point 2$b_{2}=8.2363$ [mm]$R_{2}=11.3768$ [mm]$C_{{\mathrm{NM}}_{2}}=1.262405968$$\sigma_{\max_{2}}^{\mathrm{actual}}=\sigma_{T}C_{{\mathrm{NM}}_{2}}=965.121\;\left[\mathrm{MPa}\right]$

- Specimen P1-1

P1-1$t_{0}=7.5$ [mm]$W_{0}=7.4$ [mm]F=35885.96 [N]$A_{\mathrm{current}}=46.20984$ [mm^2^]$\sigma_{T}=776.587$ [MPa]Δt=0.412963 [mm]Point 1$b_{1}=10.2741$ [mm]$R_{1}=35.14171$ [mm]
$C_{{\mathrm{NM}}_{1}}=1.168917692$

$\sigma_{\max_{1}}^{\mathrm{actual}}=\sigma_{T}C_{{\mathrm{NM}}_{1}}=907.766\;\left[\mathrm{MPa}\right]$
Point 2$b_{2}=10.2595$ [mm]$R_{2}=35.042$ [mm]
$C_{{\mathrm{NM}}_{2}}=1.169041427$

$\sigma_{\max_{2}}^{\mathrm{actual}}=\sigma_{T}C_{{\mathrm{NM}}_{2}}=907.862\;\left[\mathrm{MPa}\right]$


**Figure 9:**
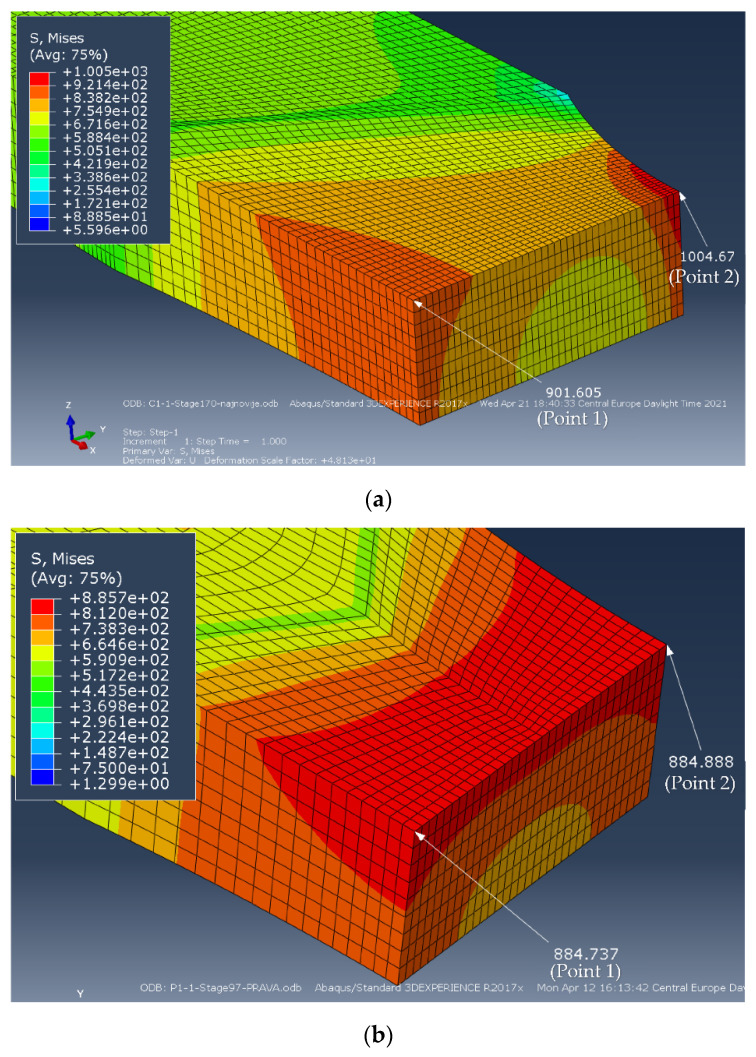
Maximum equivalent stresses in MPa at the characteristic points for specimens: (**a**) C1-1, (**b**) P1-1.

The values obtained in ABAQUS for the quarter of the specimen C1-1 and P1-1 at the characteristic points (1 and 2) are shown in Figure 9. Stress for specimen C1-1, the maximum equivalent stresses (von Misses) are:

(16) $$S_{{\mathrm{Misses}}_{1}}=901.605\;\mathrm{MPa},\;i.e.,\;S_{{\mathrm{Misses}}_{2}}=1004.67\;\mathrm{MPa}$$

For specimen P1-1, the maximum equivalent stresses (Misses) by Abaqus are:

(17) $$S_{{\mathrm{Misses}}_{1}}=884.737\;\mathrm{MPa},\;i.e.,\;S_{{\mathrm{Misses}}_{2}}=884.888\;\mathrm{MPa}$$

The equivalent stress values, obtained by ABAQUS and the stresses calculated by the formulas (10)–(15), are given in Table 5. One should notice difference between stress values in points 1 and 2 for specimen C1-1 and almost the same stress values in these two points for specimen P1-1.

**Figure 10:**
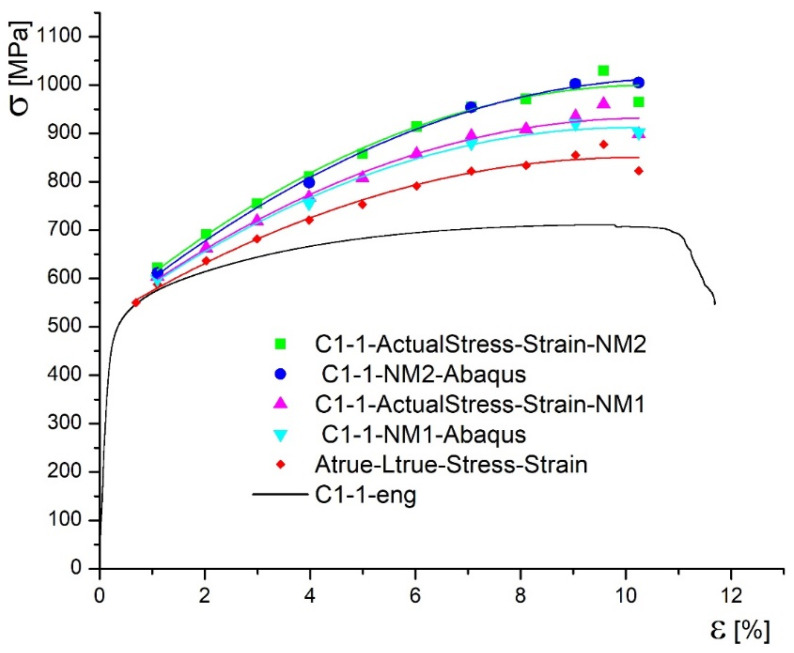
Actual stress–strain diagrams for the specimen C1-1.

**Figure 11:**
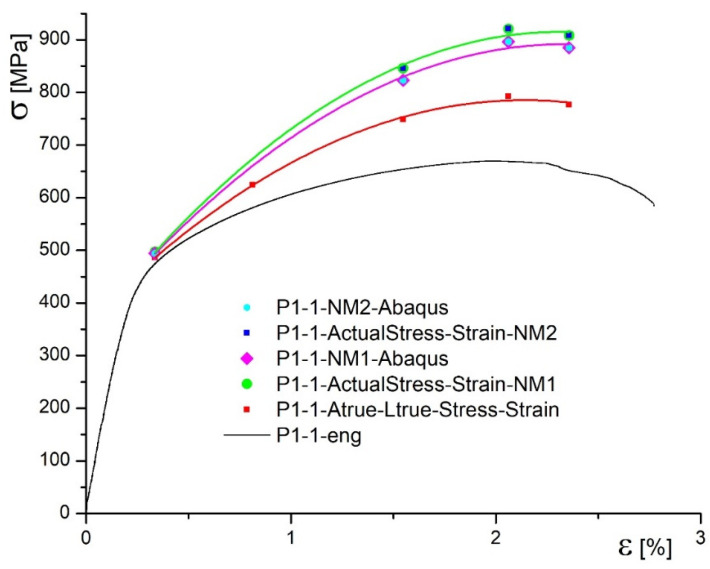
Actual stress–strain diagrams for the specimen P1-1.

In Figure 10 and Figure 11, actual, true, and engineering stress–strain diagrams are presented for the specimen C1-1 and for the specimen P1-1, respectively.

## 4. Discussion

In this research the new methodology for true stress–strain curves are applied to undermatched welded joints made of different base metals, with different geometries (cross section and groove shape). One should notice that both base metals, used in this research, are low plasticity materials, especially Armox 500T (elongation A = 8%). Therefore, using only Equations (1) and (2) for determining the true stress–strain diagram produced questionable result, since the force drop is followed by the stress drop, as shown in Figure 6 for both base metals. Thus, the real contraction, as measured by 3D DIC, should be also taken into account, providing more realistic true stress–strain curves for both base metals, also shown in Figure 6. As already mentioned, at this stage of development, one side of the specimen was actually measured, and the opposite one taken as the mirror image, while the remaining two sides are obtained by rotation. Anyhow, this issue will be tackled in future work by using at least four cameras to measure the two sides, and get the other two as mirror images. Measuring all the sides is probably too complicated, but it will be considered, as well.

Anyhow, in addition to previous, stress concentration due to geometry change should also be taken into account. Toward this end, new analytical expressions, i.e., formulas (10)–(15) have been introduced in the scope of this research, and verified by using the FEM. This was enabled by using results for strains and contraction, as obtained by DIC, to form FE models with different geometries of weld metal for different load levels, as explained in more detail in [20] using one base metal and one welded joint geometry. Here, this methodology is applied to both base metal and welded joint geometries to investigate eventual effects on actual stress–strain curves.

From Figure 10 and Figure 11 one can see that actual stresses $\sigma_{\max_{1}}^{\mathrm{actual}}$ and $\sigma_{\max_{2}}^{\mathrm{actual}}$ differ in specimen C1-1, while in the specimen P1-1 they are almost the same. Clearly, this is the effect of joint shape, since V joint (specimen C1-1) has different dimensions *b*_1_ and *b*_2_, and thus different radii of curvature *R*_1_ and *R*_2_, leading to different stress concentration factors, as well. For the specimen P1-1, difference between $\sigma_{\max_{1}}^{\mathrm{actual}}$ and $\sigma_{\max_{2}}^{\mathrm{actual}}$ is negligible due to the symmetry of joint shape (X), having approximately same values of *b*_1_ and *b*_2_, and radii of curvature, *R*_1_ and *R*_2_, leading to almost the same stress concentration factors.

It is also important to notice that differences in stresses calculated by the proposed formulas (10)–(15) and equivalent stresses obtained by Abaqus for the moment immediately before the fracture, Figure 9, do not exceed 4.10% (specimen C1-1, Table 5). With this in mind, it can be considered that the proposed formulas evaluate the actual stress correctly for different levels of undermatching and different types of weld groove, as well as different shape ratio of the specimen cross section. Therefore, it was proved here that the proposed methodology is a general one, and can be applied to different materials and welded joint geometries.

One should notice that these effects are important for undermatched welded joints, since only in this case plastic strain and stress concentration develop in the weld metal, contrary to the overmatching welded joint, where they shift to the base metal, i.e., out of the critical zones of welded joint. Anyhow, it is still important to analyze overmatching effect in future research, since it is the most often case in practice.

## 5. Conclusions

The proposed Equations (10)–(15) proved to be sound basis to determine the actual stress–strain diagrams for undermatching the welded joints made of different base metals with different welded joint geometries. Actual stresses obtained by these formulas are in good agreement with the equivalent stresses obtained by Abaqus using finite element meshes constructed according to the geometry obtained by DIC.

It can be concluded that the actual value of the tensile strength of a welded joint is far above the value obtained by the standard tensile testing, presented by engineering stress–strain curves. This difference is a consequence of cross-section contraction and stress concentration in the most deformed zone, being the weld metal in the case of undermatched welded joint.

Cross-section contraction turned out to be an important factor in the case of low plasticity material, as used in this research, since the usual formulas for “true” stress–strain curves provide questionable behavior with drop of stress after maximum tensile force is reached.

The differences in normal and equivalent stress in rectangular specimens are not significant, leading to the conclusion that the dominant effect in rectangular specimens is not triaxial stress state, but the stress concentration due to contraction.

Further analysis should use more ductile material to analyze their behavior with respect to cross-section contraction and stress concentration, as well as other types of welded joints, such as overmatching joints and different welded joint geometries, to suggest eventual corrections to the proposed formulas.

## Figures and Tables

**Table 1:** Chemical compositions of base metals.

[%]	C	Mn	Si	Ni	Cr	Mo	B	Cu	V	Ti	Zr	Al_tot_	Nb	N	P	S
1.4903	0.08–0.12	0.3–0.6	0.2–0.5	≤0.4	8–9.5	0.85–1.05	/	≤0.3	0.18–0.25	/	0.01	≤0.04	0.06–0.1	0.03–0.07	<0.02	<0.01
Armox 500T	0.32	1.2	0.4	1.8	1.0	0.7	0.005	/	/	/	/	/	/	/	0.01	0.003

**Table 2:** Chemical compositions of the filler metals.

[%]	C	Si	Mn	Cr	Ni	Mo	Nb	Cu	Ti	P	S
S Ni 6082	max 0.01	max 0.1	3.2	20.8	72.9	/	2.5	max 0.1	0.3	0.003	0.001
E 19.12.3 Nb R 26	0.02	0.9	0.7	18.0	12.0	2.7	0.4	max 0.5	/	0.02	0.02

**Table 3:** Mechanical characteristics of the base metals (BM).

BM	Yield Stress [MPa] min	Tensile Strength [MPa]	A [%] min
1.4903	450	630–830	19
Armox 500T	1250	1450–1750	8

**Table 4:** Mechanical characteristics of the filler metals (FM).

FM	Yield Stress [MPa]	Tensile Strength [MPa]	A_5_ [%]	KV [J], 20 °C
S Ni 6082	min 400	min 620	min 35	min 150
E 19.12.3 Nb R 26	min 400	min 590	min 30	min 47

**Table 5:** Comparison of the maximal stresses for the specimen C1-1 and P1-1.

Specimen	Calculated $\sigma_{max_{1}}^{actual}$	Abaqus Point 1	Difference [%]	Calculated $\sigma_{max_{2}}^{actual}$	Abaqus Point 2	Difference [%]
C1-1	898.4	901.6	0.36	965.1	1004.7	4.1
P1-1	907.8	884.7	2.6	907.9	884.9	2.6

## Data Availability

The data presented in this study are available on request from the corresponding author. The data are not publicly available due to [ongoing research].

## References

[1] Ling Y. (1996). Uniaxial true stress-strain after necking. AMP J. Technol..

[2] Bridgman P.W. (1964). Studies in Large Plastic Flow and Fracture.

[3] Ostsemin A.A. (1992). Stress in the least cross section of round and plane specimens in tension. Strength Mater..

[4] Tu S., Ren X., He J., Zhang Z. (2019). Experimental measurement of temperature-dependent equivalent stress-strain curves of a 420 MPa structural steel with axisymmetric notched tensile specimens. Eng. Fail. Anal..

[5] Scheider I., Brocks W., Cornec A. (2004). Procedure for the Determination of True Stress-Strain Curves from Tensile Tests with Rectangular Cross-Section Specimens. J. Eng. Mater. Technol..

[6] Yuan W., Zhang Z., Su Y., Qiao L., Chu W. (2012). Influence of specimen thickness with rectangular cross-section on the tensile properties of structural steels. Mater. Sci. Eng. A.

[7] Zhang Z.L., Ødegård J., Thaulow C. Novel methods for determining true stress strain curves of weldments and homogenous materials. Proceedings of the 13th European Conference on Fracture (ECF 13).

[8] Cheng C., Jie M., Chan L.C., Chow C. (2006). True stress–strain analysis on weldment of heterogeneous tailor-welded blanks—A novel approach for forming simulation. Int. J. Mech. Sci..

[9] Zhang Z.L., Hauge M., Thaulow C., Ødegard J. (2002). A notched cross weld tensile testing method for determining true stress–strain curves for weldments. Eng. Fract. Mech..

[10] Tu S., Ren X., He J., Zhang Z. (2018). A method for determining material’s equivalent stress-strain curve with any axisymmetric notched tensile specimens without Bridgman correction. Int. J. Mech. Sci..

[11] Benjamin W., Horst H. (2016). Manuela Sander, Experimental and numerical investigation of fracture in fillet welds by cross joint specimens. Procedia Struct. Integr..

[12] Fernández-Cueto M.J., Capula-Colindres S., Angeles-Herrera D., Velazquez J.C., Méndez G.T. (2018). Analysis of 3D Planar Laminations in a Welded Section of API 5L X52 Applying the Finite Element Method. Soldag. Inspeção.

[13] Javaheri E., Lubritz J., Graf B., Rethmeier M. (2019). Mechanical Properties Characterization of Welded Automotive Steels. Metals.

[14] Jiang Y., Wu Q., Zhao J., Gong J. (2019). Characterization of elastoplastic properties of dissimilar weld joint of A302/Cr5Mo using the inversion analysis. Mater. Res. Express.

[15] Younise B., Sedmak A., Milosević N., Rakin M., Medjo B. (2020). True Stress-strain Curves for HSLA Steel Weldment—Iteration Procedure Based on DIC and FEM. Procedia Struct. Integr..

[16] Younise B., Rakin M., Gubeljak N., Medjo B., Sedmak A. (2011). Numerical simulation of constraint effect on fracture initiation in welded specimens using a local damage model. Struct. Integr. Life.

[17] Younise B., Sedmak A. (2014). Micromechanical study of ductile fracture initiation and propagation on welded tensile specimen with a surface pre-crack in weld metal. Struct. Integr. Life.

[18] Sedmak S., Sedmak A. (2005). Integrity of penstock of hydroelectric power plant. Struct. Integr. Life.

[19] Jeremić L., Sedmak A., Petrovski B., Đorđević B., Sedmak S. (2020). Structural Integrity Assessment of Welded Pipeline Designed with Reduced Safety. Teh. Vjesn. Tech. Gaz..

[20] Milosevic N., Sedmak A., Martic I., Prokic-Cvetkovic R. (2021). Novel procedure to determine actual stress-strain curves. Struct. Integr. Life.

[21] Sedmak A., Milošević M., Mitrović N., Petrović A., Maneski T. (2012). Digital image correlation in experimental mechanical analysis. Struct. Integr. Life.

[22] Milosevic M., Mitrovic N., Jovicic R., Sedmak A., Maneski T., Petrovic A., Aburuga T. (2012). Measurement of Local Tensile Properties of Welded Joint Using Digital Image Correlation Method. Chem. Listy.

[23] Milosevic N., Sedmak A., Jovicic R. (2018). Analysis of strain distribution in overmatching V groove weld using digital image correlation. Procedia Struct. Integr..

[24] Milosevic M., Sedmak S., Tatic U., Mitrovic N., Hloch S., Jovičić R. (2016). Digital image correlation in analysis of stiffness in local zones of welded joints. Teh. Vjesn. Tech. Gaz..

[25] Sedmak A. (2018). Computational fracture mechanics: An overview from early efforts to recent achievements. Fatigue Fract. Eng. Mater. Struct..

[26] Banks-Sills L., Sedmak A. (2020). Linear elastic and elasto-plastic aspects of interface fracture mechanics. Struct. Integr. Life.

